# Medicine and Sociology

**Published:** 1901-11-30

**Authors:** 


### Medicine and Sociology.

A very interesting and important address on "Some of the Ethical and Sociologic Relations of the Physician to the Community," delivered by Dr. Risley, of Philadelphia, as President of the American Academy of Medicine, has just reached this country in a separate form, as a reprint from the Transactions of the Academy, and it places before us a very striking picture of the problems by which, in the United States of America, the most thoughtful members of the medical profession see themselves to be confronted, as well as of the methods by which, in their judgment, these problems may be solved. We learn from Dr. Risley that in America, as among ourselves, physicians have somehow failed to gain the general acceptance, as teachers upon all subjects capable of being elucidated by physiology, to which, on *à priori* grounds, they would seem to be entitled, and that there, as in this country, they may be seen actively lifting the burdens from other's shoulders, asking only that their own daily recurring wants should be supplied, and looking with noteworthy complacency upon the defective appreciation of their labours manifested by so large a part of every community, and even with equanimity upon the eagerness with which every new medical fad, or ism, or pathy, devised in ignorance or covetousness, is accepted by a large proportion of the laity as a new gospel of healing. In America, as in England, Dr. Risley tells us, we find in the ranks of the profession the scientific self-sacrificing physician striving to assuage pain, to heal the sick, and to prevent disease; and we find in the ranks of the charlatan the embodiment of selfishness and greed, preying, vampire-like, upon human weakness and ignorance. We could wish that the division between the two classes were always as clean and sharp as these sentences might induce an uninstructed reader to suppose; and that the influence of a diploma were always certain to destroy the spirit of charlatanism upon all those on whom it was conferred.

In his search for remedies for the evils which he describes Dr. Risley places his chief reliance upon a closer and better organisation of the medical profession, to be employed, when attained, in the great work of educating the community to a better knowledge of the meaning and value of science. He ignores all suggestions for strengthening the hands of the physician by legislative penalties or enactments, and confines himself to a sketch of the good work that might be done by the careful consideration of many great social questions by medical societies and academies, and by the careful utterance with regard to them of such pronouncements for the public guidance as science will support and experience will be found to justify. As a good illustration of the necessity for guidance of the kind, he points to the existence, in every civilised community, of a great class of "degenerates," ever seeking to reproduce their kind; a class saved from natural extinction by the altruistic impulses of modern times, and lying hopelessly beyond the reach of the defective or erroneous methods of the so-called philanthropist, whose work, in many instances, seems chiefly directed to secure a continual supply of the subjects who call for its performance. The remedy suggested by Dr. Risley is one for which public opinion in this country is not as yet prepared; but we have reached a period in the world's history at which opinion ripens quickly, and in America, on this question at least, the general tone of the address seems to justify the belief that it is in advance of our own; possibly it may be, because the social conditions there existing are such as to afford greater scope for the degenerate or the neurotic than is to be found in those of older societies. Briefly, Dr. Risley proposes the "asexualisation" of the declared criminal, and the enforcement, by public opinion, guided by adequate knowledge, or even, when necessary, by law, of restraints upon the marriage of persons who are practically certain to produce degenerate offspring. If parents, he says, can be taught that the marriage of the unfit, instead of securing them happiness, will entail upon them untold misery; if the young man can be made to understand that the silly, neurasthenic, untruthful girl will retain these qualities to the end, and will bear a progeny of like-minded children who will bring his grey hairs in sorrow to the grave; if the young woman contemplating marriage can be made to see that her reckless, drinking, thriftless lover will not reform under her gentle influence because his habits are the outward expressions of inborn vice, penetrating the whole warp and woof of his moral, physical, and mental being, then, and then only, can we hope to stem the flood of recruits annually joining the ranks of the submerged unfortunates who now fill our eleemosynary institutions, our workhouses, and our prisons.

